# Triggering of cancer cell cycle arrest by a novel scorpion venom‐derived peptide—Gonearrestide

**DOI:** 10.1111/jcmm.13745

**Published:** 2018-07-11

**Authors:** Bin Li, Peng Lyu, Xinping Xi, Lilin Ge, Ravikiran Mahadevappa, Chris Shaw, Hang Fai Kwok

**Affiliations:** ^1^ Faculty of Health Sciences University of Macau Taipa, Macau Macao; ^2^ School of Pharmacy Queen's University Belfast Belfast Northern Ireland UK; ^3^ School of Pharmacy Jiangsu Key Laboratory for Functional Substance of Chinese Medicine Nanjing University of Chinese Medicine Qixia District, Nanjing China

**Keywords:** anticancer mechanism, binding sites, MS/MS proteome, NGS transcriptome, signalling pathways, venom library

## Abstract

In this study, a novel scorpion venom‐derived peptide named Gonearrestide was identified in an in‐house constructed scorpion venom library through a combination of high‐throughput NGS transcriptome and MS/MS proteome platform. In total, 238 novel peptides were discovered from two scorpion species; and 22 peptides were selected for further study after a battery of functional prediction analysis. Following a series of bioinformatics analysis alongside with in vitro biological functional screenings, Gonearrestide was found to be a highly potent anticancer peptide which acts on a broad spectrum of human cancer cells while causing few if any observed cytotoxic effects on epithelial cells and erythrocytes. We further investigated the precise anticancer mechanism of Gonearrestide by focusing on its effects on the colorectal cancer cell line, HCT116. NGS RNA sequencing was employed to obtain full gene expression profiles in HCT116 cells, cultured in the presence and absence of Gonearrestide, to dissect signalling pathway differences. Taken together the in vitro, in vivo and ex vivo validation studies, it was proven that Gonearrestide could inhibit the growth of primary colon cancer cells and solid tumours by triggering cell cycle arrest in G1 phase through inhibition of cyclin‐dependent kinases 4 (CDK4) and up‐regulate the expression of cell cycle regulators/inhibitors—cyclin D3, p27, and p21. Furthermore, prediction of signalling pathways and potential binding sites used by Gonearrestide are also presented in this study.

## 1 INTRODUCTION

Cancer is the second most lethal disease in humans and traditional synthetic chemicals used in chemotherapy and radio‐therapeutic chemicals have extremely high cytotoxicity and cause much collateral damage to normal tissues.^1^, ^2^ Many cancer cells can develop chemo‐/radio‐resistance after a period of treatment.^3^, ^4^ Hence, the quest for new agents with lower cytotoxicity, fewer side‐effects and a lowered ability to induce drug resistance, has become an urgent goal for modern cancer therapy. The advantages of employing peptide drugs over conventional synthetic or semisynthetic organic chemicals are their efficacy at low dosage, low toxicity, innocuous metabolites, a high capacity for structural modifications and high target specificity.^5^, ^6^, ^7^, ^8^ Unlike most conventional chemotherapies, many anticancer peptides have the capacity to specifically and selectively target cancer cells and they can also be used in combination with other anticancer therapeutics, with which the observed synergistic effects have been found to improve outcomes.^9^, ^10^

Nature has always been a capable and predictable source of remarkable pharmaceutical substances with potential usage for the treatment of many diverse diseases. It has been proven that peptides/proteins are generally the major components of venoms, and many of these have either shown high potential or indeed had such realised, to defeat diseases such as bacterial/fungal infections, cardiovascular disorders, diabetes and cancers.^11^, ^12^, ^13^ However, less than 1% of the peptide/protein components of venoms have been well studied at present.^6^ One of the reasons for this is due to the fact that traditional proteomic approaches to address this problem are slow. Recently, improvements in liquid chromatography‐tandem mass spectrometry (LC‐MS/MS) have made it more accurate, sensitive and amenable to high‐throughput de novo sequencing of venom peptidomes/proteomes.^14^, ^15^, ^16^ Also, the development of next‐generation DNA sequencing technology can also facilitate higher throughput in this process and indeed is powerful enough and cost‐efficient for ultrahigh‐throughput transcriptome analysis.^17^, ^18^, ^19^, ^20^, ^21^ Some venom researchers have started to apply these two approaches, but erroneous assembly is a major limitation of next‐generation sequencing technology, while LC‐MS/MS requires an existing database of structures to validate the de novo sequencing results.^22^, ^23^, ^24^ Hence, a high‐throughput platform combining transcriptome and proteome sequencing was established in this study and employed successfully to enable large‐scale, high‐throughput identification of novel bioactive peptides in venoms.

Based on this platform, we have identified a panel of novel potential anticancer peptides in scorpion venoms. Anticancer peptide research has been performed at a low level for around 50 years now with limited success and understanding of their mechanisms of action is still in the initial stage. To reveal the potential anticancer mechanisms of candidate peptide, a recently invented transcriptome‐centric strategy was employed to predict their putative functions and targeted signalling pathways. Compared to traditional mechanistic study approaches, this was able to monitor the collective responses of all relevant genes without specific mechanistic or targeting hypotheses.^25^, ^26^, ^27^, ^28^, ^29^, ^30^, ^31^ On the other hand, although the transcriptome‐centric approach has been applied in some research, the enormous challenges in terms of data processing, storage and interpretation as well as sequencing quality control have been huge limitations which have hindered the translation from sequence data to clinical practice, but a few studies have succeeded in proving the concept.^32^, ^33^, ^34^, ^35^ Hence, here, we performed related in vitro*,* in vivo and ex vivo experiments to lend further substance to the validation of this approach.

In this study, we initially isolated a panel of potential anticancer peptides from venoms using this state‐of‐the‐art high‐throughput platform. Subsequently, a transcriptome‐centric method was applied to address and to reveal the putative anticancer mechanism of the lead peptide candidate, followed by in vitro, in vivo and ex vivo experimental validation. In addition, we hypothesized as to the involvement of specific signalling pathways and potential binding sites through bioinformatic analyses and use of 3D modelling construction software. Finally, one peptide candidate (Gonearrestide) was identified to affect cancer cell proliferation in vitro and reduce tumour growth in vivo, while negligible cytotoxicity was observed on normal human epithelial cells and erythrocytes. These current data suggest that Gonearrestide has a high potential for development as an anticancer drug. In addition, the findings presented here have further validated the ever‐increasing potential of this high‐throughput platform and transcriptome‐centric mechanistic study strategy to reveal additional novel anticancer peptide drug candidates.

## 2 MATERIALS AND METHODS

### 2.1 Acquisition of scorpion venom

Venoms from the scorpions, *Androctonus mauritanicus* (AMa) and *Androctonus australis* (Egypt) (AAu), were purchased from Latoxan, Valence, France. The lyophilized venoms were stored at −20°C prior to use.

### 2.2 Transcriptomic procedures

Five mg sample from each lyophilized scorpion venom was dissolved in 1 mL of cell lysis buffer (Thermo Fisher Scientific). Polyadenylated mRNA was extracted by use of a Dynabeads^®^ mRNA DIRECT^™^ Kit (Ambion by Life Tech), then subjected to a cDNA library construction procedure using a NEBNext^®^ Ultra^™^ Directional RNA Library Prep Kit for Illumina^®^ (New England BioLabs Inc.). According to the instructions, the cDNA library construction consisted of several main steps. First of all the mRNA fragmentation was achieved by incubating at 94°C with random primers. Then the RNA fragments were subjected to first strand cDNA and second strand cDNA syntheses. After purification with 1.8X Agencourt AMPure XP beads, end repair of the cDNA library was performed by incubating with the end repair reaction buffer and the nucleotide A was added to the 3′ end of the DNA fragments to avoid self‐ ligation. Therefore, the adapters with the nucleotide T at the 3′ end were ligated to the DNA fragments. At last, the DNA fragments with adapters were enriched by PCR reactions and purified by AMPure XP beads. The quality of the cDNA library was verified using an Agilent 2100 Bioanalyzer (Agilent Technologies) with an Agilent DNA 1000 kit (Agilent Technologies). The quantity of the cDNA library was validated by qPCR with a KAPA SYBR^®^ FAST qPCR kit (KAPA Biosystems). The cDNA library was then loaded into a flow cell with oligos complementary to the adapters to generate clusters through bridge amplification. Finally, the transcriptome was obtained by performing RNA Sequencing on the Illumina MiSeq platform. The raw data obtained from the Miseq platform were analysed as follows. Firstly, the index primers used to identify different samples were removed. Secondly, the data were transferred to the program FastQC 0.1.0.1 to filter out the reads with low quality and less than 25 nucleotides. The filtered data were saved as fastq files. At last, the clean reads were de novo assembled using Trinity 2.0.6 software to obtain the transcriptome with default parameters.

### 2.3 Proteomic procedures

A second set of 5 mg lyophilized scorpion venom sample was dissolved in phosphate buffer (50 mmol/L, pH 7, containing 0.15 mol/L NaCl), and subjected to AKTA Avant 150 (GE Healthcare Life Science) fractionation using a Superose^®^ 12 10/300 GL column (Sigma Aldrich) to filter out large proteins with molecular masses higher than 10 KD. After this, Tris (2‐chloroethyl) phosphate (TCEP) was used to reduce disulphide bonds, and iodoacetamide (IAA) was used to alkylate free cysteine residues. At last, desalting was performed using PierceTM C18 Spin Tips (Thermo Scientific). Proteomics was performed using the Q Exactive^™^ Hybrid Quadrupole‐Orbitrap Mass Spectrometer (Thermo Scientific) with a nano‐LC system. Samples were loaded onto the Acclaim pepMap100, 75 μm × 2 cm C18 trap column (Thermo Scientific) using LC‐MS water (containing 2% ACN, 0.1% formic acid). Samples were trapped onto the column at 6 μL/min for 5 minutes for pre‐concentration, then the trap column was connected to an EASY‐Spray column, 15 cm × 75 μm ID, 3 μm‐C18 particle sizes (Thermo Scientific) by a 10 port valve automatically. Eluting was performed at a flow rate of 300 nL/min with the gradient mobile phase buffer (Mobile phase buffer A: LC‐MS water, 2% ACN, and 0.1% formic acid; Mobile phase buffer B: ACN, 2% LC‐MS water and 0.1% formic acid). Eluting peptide cations were converted to gas‐phase ions by electrospray ionization with a spray voltage of 1.8 kV and capillary temperature of 300°C. Full MS scans of peptide precursors from 300 to 1800 m/z were performed at 70 K resolution, while the AGC target was set to 3 × 10^6^ ion counts with a max injection time of 200 ms. MS2 was performed by an isolation window at 2 m/z with the quadrupole, high energy collision (HCD) fragmentation with normalized collision energy of 27. The resolution was 17.5 K, while ion count target was set to 10^6^ and the max injection time was 110 ms. The top 20 precursor ions were selected in each cycle and only those precursors with a charge state +2 to +5 were isolated for MS2 fragmentation. The fixed first mass of MS2 spectrum range started at 100 m/z. The C‐trap under fill ratio was 2%, which converted to the intensity threshold of 1.7 × 10^4^ for the precursor selection. The dynamic exclusion duration was set to 20 seconds and isotope exclusion was turned on. The LC‐MS/MS raw data were then transferred to PEAKS (version7.5) software for de novo sequencing with default parameters.

### 2.4 Comparison between transcriptome and proteome

The software, PEAKS (version8.0) (Bioinformatics Solutions Inc., Waterloo, ON, Canada), was used to interrogate the transcriptome database with parameters set as: precursor ion mass tolerance, 3 ppm; fragment ion mass tolerance, 0.01 Da; no enzymes employed; a variety of post‐translational modifications (PTMs) including cysteine carbamidomethylation and oxidation, were used and false discovery rate (FDR) was at ≤1%. The peptides identified high confidence and accuracy, constituted the prospective venom‐derived peptide libraries.

### 2.5 Molecular cloning procedures

A third set of 5 mg lyophilized scorpion venom sample was dissolved in 1 mL of cell lysis/mRNA protection buffer, and polyadenylated mRNA was isolated from each by magnetic oligo‐dT beads as described by the manufacturer (Thermo Fisher Scientific). The isolated mRNA was subjected to 5′‐ and 3′‐rapid amplification of cDNA ends (RACE) procedures using a SMART‐RACE kit (Clontech, UK). The 3′‐RACE reactions were purified and cloned using a TOPO^®^ TA Cloning^®^ Kit (Invitrogen) and sequenced using an ABI 3100 automated sequencer. The nucleic acid sequences were isolated by employing the primers below, which were designed to highly‐conserved domains of the 5′‐untranslated regions of previously‐characterized venom peptide cDNAs from scorpions: (*i*) Primer AMa_Mauri1 (5′‐CATTACTATGTTGATTGTCGATGAAGTC‐3′); (*ii*) Primer AAm _H2 (5′‐AGTTTGGCACTTCTCTTCGTGAC‐3′); (*iii*) Primer And_Uni1 (5′‐TAGWCCTGSTRGTYARTCCGA‐3′).

### 2.6 Novel venom‐derived peptide library construction and peptide synthesis

The prospective novel venom‐derived peptide libraries were subjected to BLAST analyses against the NCBI non‐redundant database and the peptide sequences which produced hits with the sequences on this were then subjected to Pfamscan online searching to identify whether the sequences aligned to any reported toxins. The peptide sequences reported before were removed manually during this step. The novel peptides were manually filtered by removing very short sequences and finally, the venom‐derived peptide library was constructed with all the peptide sequences selected as above. The selected peptide regions were chemically‐synthesized by solid phase peptide synthesis (SPPS) technology.

### 2.7 Cell culture and potential anticancer peptide screening

Cancer cell lines were maintained in their culture medium with 10% (v/v) foetal bovine serum (FBS) and 1% (v/v) penicillin/streptomycin. All cells were seeded into 75 cm^2^ culture flasks (Falcon, Fisher Scientific), and cultured in an incubator under 5% CO_2_/95% air at 37°C. The anti‐proliferative effects of these novel peptides on human cancer cells were measured by employing MTT cell viability assays. Peptides were screened at a concentration of 10 μmol/L with various cell lines: Pancreatic cancer cell lines MIA Paca‐2, PANC‐1, AsPC‐1; Colon cancer cell lines HCT116, DLD‐1, Hke3, Dks8; Breast cancer cell lines MCF‐7, MDA‐MB‐231; Bladder cancer cell line HT‐1376; Human retinal epithelial cancer cell line ARPE‐19; Brain glioma cell line U‐251. Two different normal epithelial human cell lines (colon cell line FHC and breast cell line MCF 10A) were also employed in the screening assays. Peptides with anti‐proliferative activities were then selected and further studied in a dose‐dependent manner in the concentration range from 250 to 1 μmol/L.

### 2.8 Haemolysis assay

Human erythrocytes were washed with PBS until the supernatant was clear, and then samples of a 4% (v/v) suspension were treated with peptide concentrations ranging from 1 to 250 μmol/L at 37°C for 1 hour. Lysis of cells was assessed by haemoglobin release, measured by optical density changes at λ550 nm, and calculated compared to positive controls. Positive control groups and negative control groups were treated with equal volumes of Triton X‐100 and PBS instead of peptide, respectively.

### 2.9 LDH assay

Cells were treated with different peptide concentrations from 250 to 10 μmol/L for 24 hours. LDH Cytotoxicity Assay Kit (Cayman, USA) was then employed to detect the cytotoxicity of peptides according to the manufacturer's instructions. The supernatants were transferred, mixed with the reaction buffer and LDH release was detected using a microplate reader set at 492 nm and calculated compared to positive controls. Positive control groups and negative control groups were treated with equal volumes of Triton X‐100 and PBS instead of peptide, respectively.

### 2.10 Apoptosis assay (phosphatidylserine exstrophy detection)

Cells were treated with peptides at the concentration of 250 μmol/L for 24 hours, after which the Alexa Fluor 488 annexin V/Dead Cell Apoptosis Kit (Invitrogen) was employed to detect apoptosis according to the manufacturer's instructions along with use of a flow cytometer BD Accuri^™^ C6. The blank control group was treated with an equal volume of PBS.

### 2.11 Proliferation curve generated through an IncuCyte live cell imaging system

Cells were seeded into 6‐well plates and treated with peptides at 250 μmol/L for 24 hours, after which the IncuCyte ZOOM^™^ Continuous Live‐cell Imaging & Analysis System was employed to record cell growth for three days. The instrument can automatically generate cell growth curves, which are calculated by the cell area attached to the bottom of each well according to the images taken by the instrument every 2 hours. Blank control group was treated with equal volumes of PBS.

### 2.12 RNA sequencing reveals signalling pathways altered by peptide treatment

Samples were prepared by treating cells with Gonearrestide at 250 μmol/L for 24 hours. Blank control groups and negative control groups were treated with equal volumes of PBS and negative control peptide, respectively. For each group, two replicates were employed. Samples were collected and total RNA was extracted, DNA library preparation was performed as previously described under “Transcriptomics procedures”. Transcriptome sequencing and analysis procedures were performed as follows: TruSeq RNA‐seq libraries were sequenced on the Illumina HiSeq2000 platform. The raw reads were mapped to the human genome (version: GRCh38, http://ftp://ftp.ensembl.org/pub/release-87/fasta/homo_sapiens/dna//Homo_sapiens.GRCh38.dna.primary_assembly.fa.gz) using the splice aligner tophat2 (v2.1.1) with default parameters.^36^ Gene expression analysis was performed using cuffdiff (v2.2.1) from tuxedo pipeline.^37^ CummerBund 2 (v0.1.3, R package) from tuxedo pipeline was used to normalize the data sets and calculate the fold changes and their statistical significance on the basis of two independent biological replicates for each set of conditions. Genes with fold changes of 1.5 (log2fold change: absolute (0.6)) and *q*_value cut‐off of <0.05, were considered as significantly differentially expressed. Further GO enrichment and pathway visualization of differentially expressed genes was performed by the DAVID online tool (https://david.ncifcrf.gov) and KEGG mapper (http://www.genome.jp/kegg/tool/map_pathway2.html), respectively.

### 2.13 Detection of cell cycle status through use of flow cytometry

Cells were treated with peptides at 250 μmol/L for 24 hours, after which cells were collected and fixed with cold 70% ethanol overnight. The next day, cells were washed with PBS and stained with staining working solution (500 μL PBS containing 50 μg/mL PI, 100 μg/mL RNase A, and 0.2% Triton X‐100) for 5 minutes at room temperature. Density of cells in each cell cycle phase was recorded by flow cytometry using a BD Accuri^™^ C6. The blank control group was treated with an equal volume of PBS.

### 2.14 Identification of involved proteins in the cell cycle process through Western blotting

Cells were treated with peptides at 250 μmol/L for 24 hours. The blank control group was treated with an equal volume of PBS, and regarded as time‐point 0 hour. Cells treated with peptides were then collected at different time‐points (treated after 2, 6, 12, 24, 48, 72 hours) and washed with cold PBS. RIPA buffer (150 mmol/L NaCl, 5 mmol/L EDTA, 50 mmol/L Tris‐HCl, 1% NP‐40, 0.5% sodium deoxycholate, 0.1%SDS) was used to lyse cells for 30 minutes on ice, after which cells were centrifuged at 20 000× g for another 30 minutes in a 4°C pre‐cooled centrifuge. The supernatants were stored at −80°C prior to use. Equal amounts of protein samples at each time‐point, including those from the blank control group, were subjected to SDS‐PAGE and then transferred to a membrane. Membranes were then blocked with 5% non‐fat dry milk in TBST (0.1% Tween) for 2 hour at room temperature, and then incubated with primary antibodies (against Cyclin D1, Cyclin D3, CDK2, CDK4, CDK6, p18, p21, p27) at 4°C overnight. The next day, TBST (0.1% Tween) was used to wash membranes three times, after which secondary HRP conjugated antimouse/anti‐rabbit antibody was incubated with membranes for 1 hour at room temperature. After washing with TBST (0.1% Tween) another three times, Immobilon Western HRP Substrate was employed for chemiluminescent detection in Western blots.

### 2.15 Characterization of cellular location of peptide through use of confocal microscopy

Cells were cultured in an 8‐chamber slide and treated with fluorescently labelled peptides for 1 hour. After this, cells were stained with CellMask Plasma Membrane Stains (Invitrogen) and Hoechst dye (Thermo Fisher) according to the manufacturer's instructions, followed by washing with PBS and fixation with 3.75% formaldehyde. The locations of peptides affecting cells were recorded by confocal laser scanning microscopy.

### 2.16 In vivo anticancer activity of the peptide

Nude Mice used in this study were obtained from the Animal Facility in the Faculty of Health Sciences at the University of Macau (UM). All the experimental protocols were conducted according to the UM Ethics Committee for Animal Experiments with the approved application UMARE‐035‐2016.

In vivo anticancer activity of Gonearrestide was evaluated in an HCT116 colon cancer xenograft model. 1 x 10^6^ HCT116 colon cancer cells were xenotransplanted by subcutaneous injection (s. c.) into the flanks of 4 week‐old female nude mice. After 3 days of implantation, mice were then divided into four groups: Group 1 (n = 5) was treated with Gonearrestide at a high concentration (100 μg/tumour) by peritumoral (p. t.) injection for another 2 weeks; Group 2 (n = 5) was treated with Gonearrestide at low concentration (50 μg/tumour) while Groups 3 and 4 (n = 5) were treated with an equal volume of PBS and negative control peptide, respectively. Tumour volume measurement was performed during 2 weeks of treatment. To determine tumour volume by use of an external caliper, the greatest longitudinal diameter (length) and the greatest transverse diameter (width) were determined. Tumour volumes, based on caliper measurements, were calculated by the modified ellipsoidal formula^38^, ^39^: Tumour volume = 1/2(length × width^2^). At the end of the experiment, tumours were removed under anaesthesia and mice were then euthanized. Tumours were snap‐frozen for further histological analysis. On the one hand, tumours were fixed and cut into slides. Immunohistochemical staining of tumour slides were performed by de‐paraffinizing in xylene and rehydrating in graded ethanol, followed by incubation in sodium citrate buffer (pH 6.0) for high‐pressure antigen retrieval. Afterwards, slides were incubated in 3% hydrogen peroxide to block endogenous peroxidase activity, and incubated with cell cycle checkpoint antibody CDK4 at 4°C overnight. On the second day, the slides were incubated with secondary antibody and subjected to diaminobenzidine staining. After counterstaining with 20% haematoxylin, slides were dehydrated and mounted on cover slips. The IHC‐stained tissue sections were reviewed under a microscope. In addition, mRNA and proteins were isolated from frozen tumours for qPCR and Western blot analysis. The procedures were for both techniques were described before.

### 2.17 Statistical analysis

Statistical analysis was performed using GraphPad Prism 6 software. *P* values were calculated by student's t‐test from the mean values of the indicated data. Significant differences were marked with asterisks (*P < .05; **P < .01; ***P < .001; ****P < .0001).

## 3 RESULTS

### 3.1 Novel venom‐derived peptide library construction through combining transcriptome and proteome analyses

#### 3.1.1 RNA sequencing with next‐generation sequencing technology

In total, there were 514 974 and 625, 389 raw reads generated for the scorpion species, *Androctonus mauritanicus* (AMa) and *Androctonus australis* (Egypt) (AAu), respectively. As these two scorpion species do not have their genomes assembled so far, the transcriptome data were de novo assembled by use of Trinity software. Consequently, there were 112 235 and 46 360 nucleic acid sequences, respectively, obtained for each species (Table 1A). The transcripts were saved as Fastq files (S1 and 2).

**Table 1:** (A) Summary of Transcriptome and Proteome data. (B) Summary of up and down‐regulated genes between each group in RNA sequencing data of HCT116 cells treated with/without Gonearrestide

(A)				
Scorpion species	*Androctonus mauritanicus*		*Androctonus australis (Egypt)*	
Transcriptome de novo assemble				
Raw reads	514 974		625 389	
Nucleic acids sequences	112 235		46 360	
Proteome de novo sequencing				
Spectrums	20 606		16 205	
Peptide fragments	9225		7464	
Filtered peptide fragments^a^	3346		2393	
Comparison between Transcriptome and Proteome				
Database	Scorpion	Transcriptome	Scorpion	Transcriptome
Spectrums	872	3398	646	2516
Peptide fragments	351	1176	267	924
Peptides	67	128	63	110

(B)		
Comparison	Up‐regulated genes	Down‐regulated genes
HCT116_BC vs HCT116_Gonearrestide	1906	2032
HCT116_NC vs HCT116_Gonearrestide	1835	2510
HCT116_BC vs HCT116_NC	Not significant	Not significant

a Filtration parameter: ALC (average local confidence) > 50%.

#### 3.1.2 Isolation and de novo sequencing of proteomes through use of a highly accurate and sensitive LC‐MS/MS system

The raw spectra generated from the AMa venom contained 20 606 peptides fragments. After alignment, the remaining 9225 peptide fragments obtained were filtered with the filtration parameter setting of an average local confidence of more than 50%. The de novo sequencing results including 3346 peptide fragments were saved as Excel files (S3; Table 1A). Similarly, from the AAu venom, we identified 2393 different peptide fragments after filtered from 7464 peptide fragments (S4; Table 1A).

#### 3.1.3 Comparison between respective transcriptomes and proteomes

In AMa transcriptome and proteome analysis, there were 1176 peptide fragments in the overlapping region between transcriptome and proteome databases for the two species, which aligned to 128 peptides (Table 1A). With the same method, 110 peptides were aligned from 924 peptide fragments in AAu transcriptome and proteome analysis (Table 1A). The overlapping peptides were regarded as the prospective venom‐derived peptide libraries and saved as Excel files (S5).

To further validate our findings, we also compared AMa and AAu proteomes with an online scorpion protein database (UniProt). In total, there were 130 peptides matched with UniProt database, which indicated the robustness of our data.

#### 3.1.4 Molecular cloning

There were eight peptide sequences isolated through the molecular cloning approach, including three novel peptide sequences. All these eight peptide sequences were found in the overlapping region of the transcriptome and the proteome results, which validated the new high‐throughput approach. The nucleotide and translated open‐reading frames of the three novel sequences were saved (Figure S1).

#### 3.1.5 Novel venom‐derived peptide library construction and peptide synthesis

The overlapping regions described previously were subjected to a variety of bioinformatic analyses and the filtered results were saved (Figure S2). As a result, novel venom‐derived peptide libraries with 41 and 30 peptides were obtained for scorpion species AMa and AAu, respectively (S5). Twenty‐two peptide sequences were initially selected to synthesize for use in further analyses (Table S1).

### 3.2 Biological functional screening of the synthesized peptides

#### 3.2.1 Cell anti‐proliferative assay

**Figure 1:**
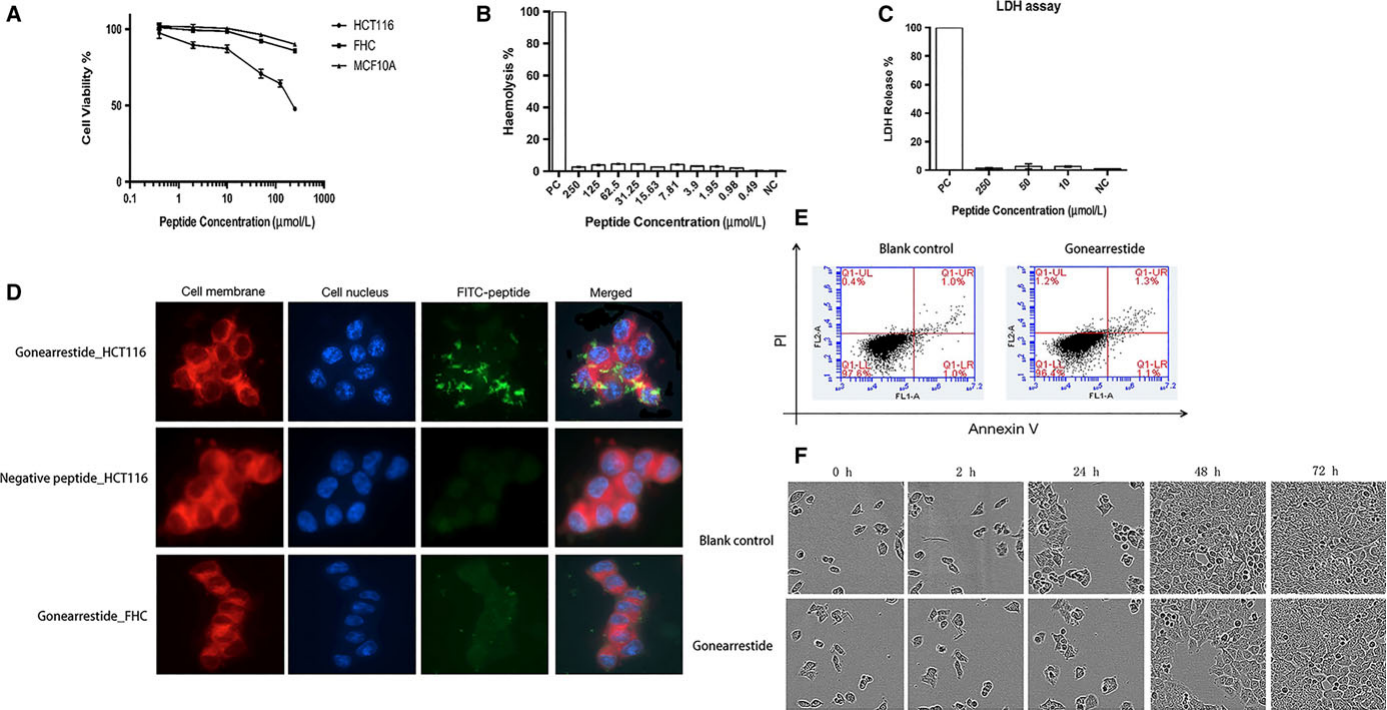
Anti‐proliferative of Gonearrestide. A, Dose‐dependent anti‐proliferative effects of Gonearrestide treated on human cancer cell lines HCT116 and human normal cell lines MCF10A, FHC after 24 h of incubation (n = 15). The growth inhibition of cells at each peptide concentration was calculated as a percentage of the growth observed in controls, which was treated as 100%. B, Haemolytic activity of Gonearrestide with gradient concentrations on human red blood cells. Haemolysis was calculated as a percentage compared with positive control (add Triton X‐100), which was treated as positive control (n = 15). C, LDH release of Gonearrestide with gradient concentrations on cancer cell HCT116. LDH release was calculated as a percentage compared with positive control (add Triton X‐100), which was treated as 100% (n = 15). D, Cellular location of FITC‐labelled peptide binding on human cell lines. (Confocal microscope with 63X/oil magnification) E, Apoptosis result of Gonearrestide on HCT116 using flow cytometry. Blank control group was cells without adding peptide. F, Inhibition effect of Gonearrestide working on human colon cancer cell line HCT116 generated by IncuCyte live cell imaging system. Blank control group was cells treated with equal amount of PBS (n = 15)

In total, 22 peptides were screened with 19 cell lines to identify their potential anti‐proliferative activities, after which four peptides (Peptides 4, 6, 10 and 13) were found to inhibit the proliferation of human colon cancer cell lines HCT116, DLD‐1, Hke3, Dks8 and the human glioma cell line U‐251. Two types of normal human epithelial cell lines, including the colon cell line FHC and the breast cell line MCF 10A, were also employed here and it was found that the peptides had negligible effects on their proliferation even at highest concentrations. Peptide 13 (Gonearrestide, the amino acid sequence was shown in Table S1) was found to have the most potent activity on human colon cancer cell line HCT116, thus it was chosen as a lead peptide for further investigation. The does‐dependent anti‐proliferation effect of Gonearrestide was shown in Figure 1A.

#### 3.2.2 Haemolysis assay

This demonstrated that Gonearrestide had negligible cytotoxicity on human erythrocytes with a haemolytic activity below 5% (positive control was regarded as 100%), indicating that Gonearrestide was a worthy candidate for further study with the view to its clinic application (Figure 1B).

### 3.3 Biological functions validation of Gonearrestide in vitro

#### 3.3.1 LDH assay

Lactate dehydrogenase (LDH) is released when cell membrane integrity is compromised. In this assay, Gonearrestide did not induce LDH release from HCT116 cells after 24 hours of incubation. This result indicated that Gonearrestide did not disrupt the cell membrane and did not induce cell death. At the same time, the LDH results were consistent with the RNA sequencing data along with the combination of MTT results. Taken together, these data confirmed that Gonearrestide affected cancer cell proliferation through inhibition of growth (Figure 1C).

#### 3.3.2 Determination of cellular location of peptide through use of confocal microscopy

We further investigated the cellular location of Gonearrestide by incubating FITC‐labelled Gonearrestide (250 μmol/L) with HCT116. Localization of Gonearrestide within the cell should result in green fluorescence signals from the FITC‐labelled peptide. As shown in Figure 1D, Gonearrestide treated HCT116 cells showed strong green fluorescence signal on the cell membrane of HCT116. In contrast with the normal human epithelial cell group, there was almost no signal detected. In another set of control experiment, we also found that incubation of negative peptide to HCT116 cell lines did not show green fluorescent in the cell membrane at all. These data clearly indicated that Gonearrestide was specifically localized on the cancer cell membrane only. On the other hand, green fluorescence signal was not detected in cytoplasm or nuclear of the cell, suggesting that Gonearrestide did not enter into the cell compartments. Taken together, it demonstrated that Gonearrestide was specifically bound to the cancer cell membrane, and induced subsequent reactions via membrane‐related signalling pathways which will further be hypothesized and analysed in our “cell‐peptide” RNA sequencing experiments.

#### 3.3.3 Apoptosis assay (Phosphatidylserine exstrophy detection)

Annexin V and PI staining along with flow cytometry can differentiate individual cells into different stages. The results of these experiments were shown in Figure 1E. The data showed that Gonearrestide did not induce apoptosis of HCT116 cells when compared to blank controls—a finding which was also consistent with our RNA sequencing data in this study.

#### 3.3.4 Proliferation curve generated through IncuCyte live cell imaging system

As shown in Figure 1F, the proliferation of HCT116 cells treated with Gonearrestide was much slower than that observed for the blank controls. The results proved that Gonearrestide could inhibit cancer cell growth which again was consistent with our RNA sequencing data in this study.

### 3.4 RNA sequencing revealed the biological function and signalling pathways altered by peptide treatment

As Gonearrestide had the best activity on the cancer cell line HCT116 with no obvious cytotoxicity on normal human cell lines and erythrocytes, it was chosen for further experiments to identify its molecular mechanism of action. There were three groups employed in this assay: HCT116 treated with Gonearrestide (HCT116_Gonearrestide), HCT116 treated with a negative control peptide (HCT116_NC) and HCT116 treated with PBS (HCT116_BC). The negative control peptide was one of the previously identified peptides which showed no anticancer cell activity.

**Figure 2:**
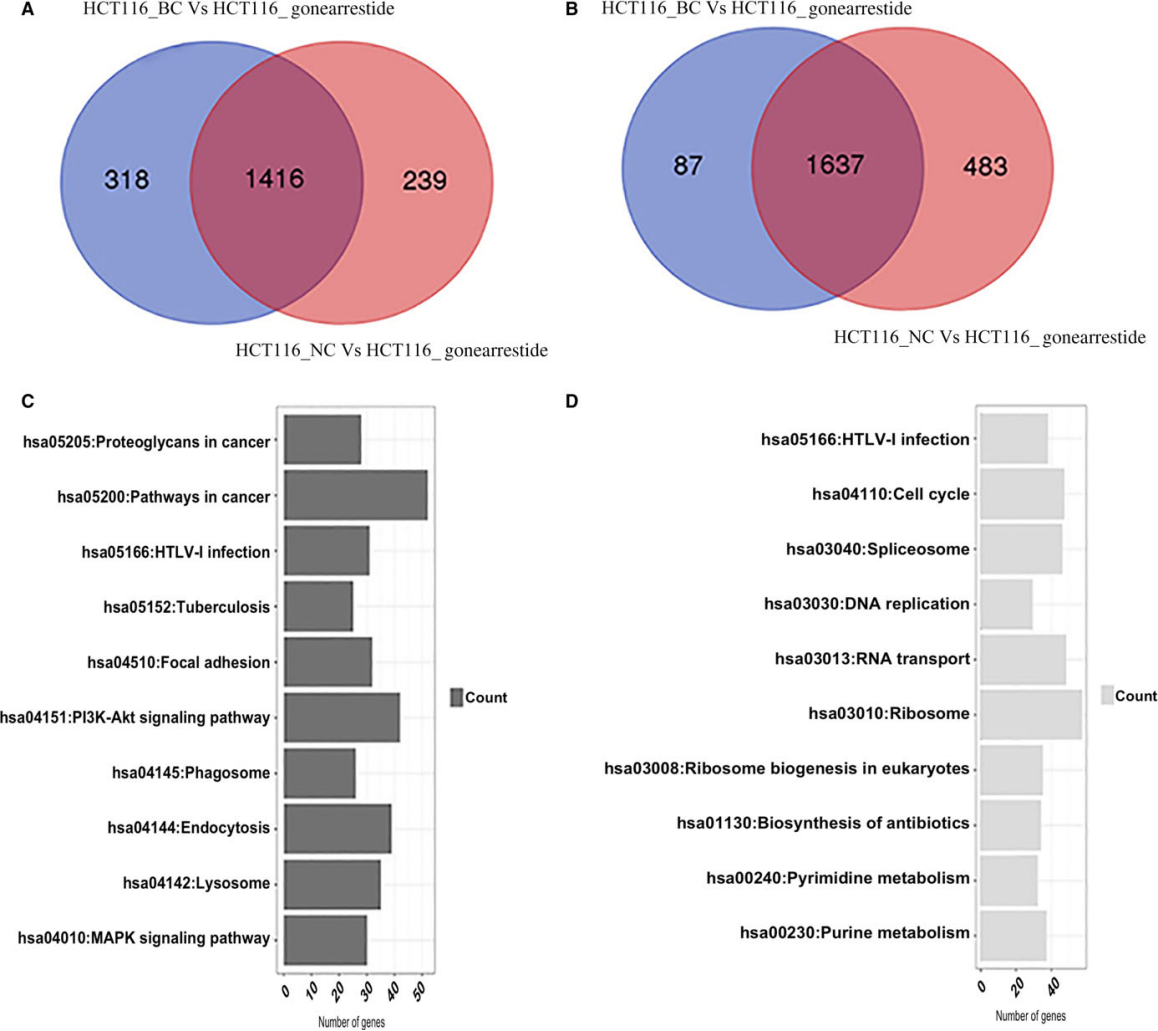
Comparison results and function/pathway annotation of HCT116_BC vs HCT116_Gonearrestide set and HCT116_NC vs HCT116_Gonearrestide set. A, Up‐regulated genes. B, Down‐regulated genes. C, Top 10 up‐regulated pathways. D, Top 10 down‐regulated pathways

Approximately 40 million reads were generated per sample and around 90% of these were aligned to the human genome with a correlation value of 0.9 between and across replicates (Figure S3). The top 500 variable genes (based on standard deviation) demonstrated that the replicates were consistent but slight differences were present between the blank control and the negative peptide groups (Figure S4). Genes were determined as being significantly differentially expressed (DEG) based on a twofold log change and a q‐value cut‐off of absolute (0.6) and 0.05, respectively (S6). After filtration with the fold change and statistical significance, the remaining significantly differentially expressed genes were shown in Table 1B. There were around 2000 genes up‐ or down‐regulated after Gonearrestide treatment, while the differences between the blank control and negative peptide groups were not significant. From the comparison of the HCT116_BC vs the HCT116_Gonearrestide data sets and the HCT116_NC vs the HCT116_Gonearrestide data sets, 1416 common genes were found to be up‐regulated, while 1637 common genes were down‐regulated (Figure 2A and B). The top 10 pathways (*P* value < .05, tool: DAVID) enriched under common genes (1416, 1637) have been listed (Figure 2C and D).

**Figure 3:**
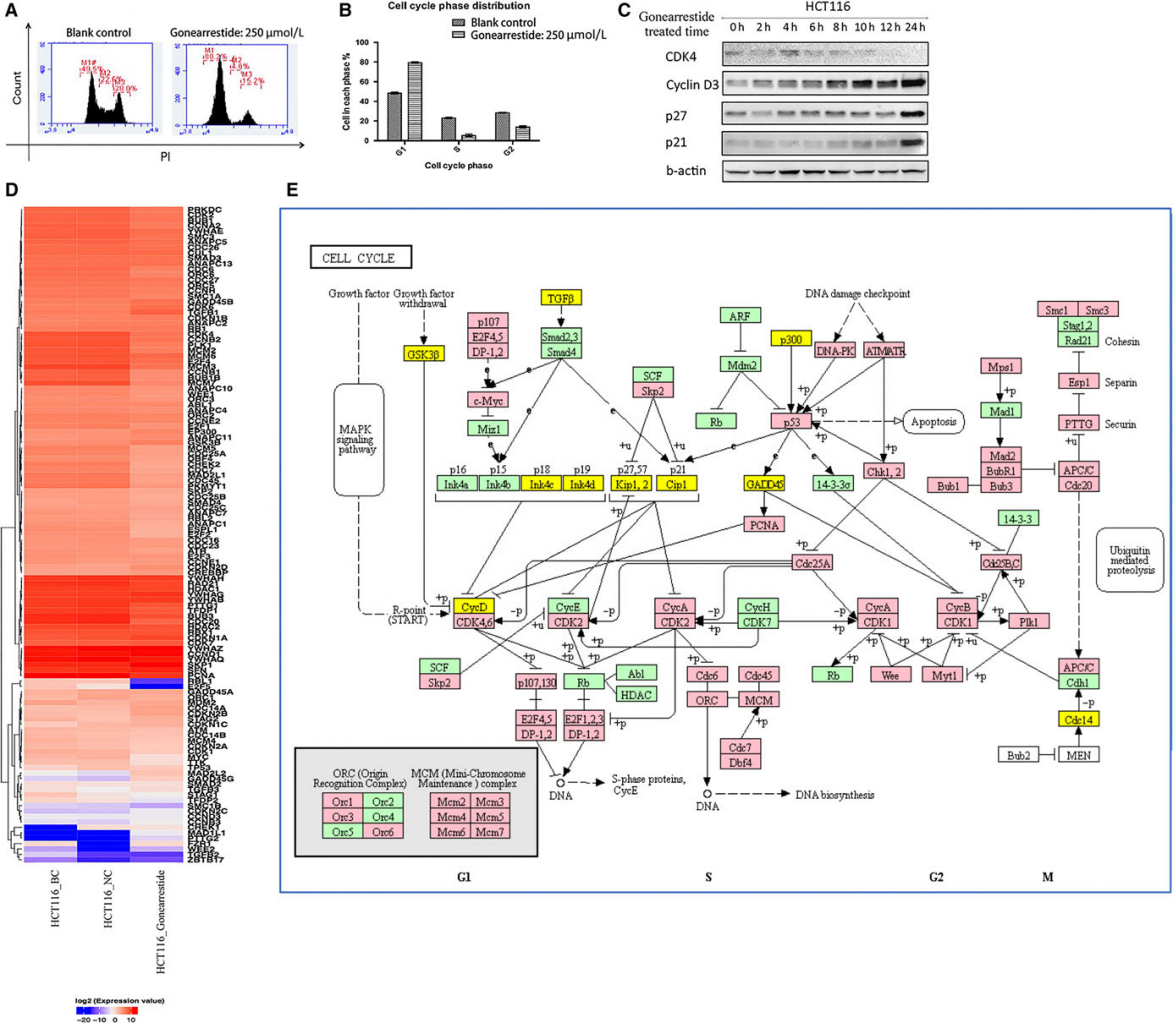
Cell cycle assay results. A, Cell cycle phase distribution of Gonearrestide on human colon cancer cell line HCT116 after 24 h of incubation using flow cytometry. B, Calculated percentage of cells in each phase (n = 9). C, Cell cycle‐related proteins regulated by Gonearrestide on HCT116. Time‐point “0 h” was blank control group treated with equal amount of PBS. D, Heat map of cell cycle‐related genes. E, Cell cycle checkpoint control‐related pathways (yellow indicating up‐regulated genes and red indicating down‐regulated genes)

As shown in Figure 2C, the up‐regulated functions and pathways included proteoglycans in cancer, focal adhesion, MAPK signalling pathway and dysregulated pathways included spliceosome, RNA transport and ribosome. Ribosome biogenesis in eukaryotes, plays an important role in cell proliferation.^40^, ^41^, ^42^, ^43^, ^44^, ^45^, ^46^, ^47^ On the other hand, the PI3K‐Akt signalling pathway, cell cycle, DNA replication, pyrimidine metabolism and purine metabolism, are all closely related to cell cycle progression. Hence, we hypothesized that Gonearrestide could affect the proliferation of HCT116 cancer cells through cell cycle arrest effects. Heat maps of cell cycle‐related genes and cell cycle checkpoint control‐relevant pathways were generated and shown in Figure 3D and 3E. As most of the pathways annotated to the functions of cell proliferation and cell cycle, in vitro and in vivo biological assays were then employed to verify the hypothesis. In addition, current results showed that peptide treatment did not cause cell death or apoptosis, so LDH and apoptosis assays were also employed to validate the hypothesis from another point of view. In addition, there were still some other functions and pathways involved of note as shown in Figure 2C, such as phagosome, endocytosis and lysosome, which are all related to membrane reactions. Hence, we hypothesized that Gonearrestide worked through initial combination with cancer cell membranes, then produced subsequent effects. Based on this, confocal imaging experiments were designed and conducted as described in Figure 1D.

### 3.5 In vitro cell cycle arrest effect on Gonearrestide

#### 3.5.1 Detection of cell cycle phase through flow cytometry

Cell cycle phase distribution of HCT116 cells treated with/without Gonearrestide was shown in Figure 3A and B. These data demonstrated that Gonearrestide arrested cancer cell cycle in G1 phase, and was consistent with our findings in Figure 2D which showed the cell cycle signals were actually down‐regulated after Gonearrestide treatment.

#### 3.5.2 Identification of proteins involved in cell cycle processes by Western blotting

As Gonearrestide could arrest HCT116 cells in G1 phase, G1/S checkpoint protein antibodies were employed to identify the regulatory proteins involved, including cyclin D1, cyclin D3, CDK2, CDK4, CDK6, p18, p21 and p27. Western blot results are shown in Figure 3C, and the data demonstrated that Gonearrestide worked through inhibiting the cyclin‐dependent kinase 4 (CDK4), and up‐regulating the expression of the cell cycle protein regulators/inhibitors, cyclin D3, p27, p21. These results were again consistent with the RNA sequencing data and also matched with our predicted pathways.

### 3.6 In vivo anticancer activity validation of Gonearrestide

**Figure 4:**
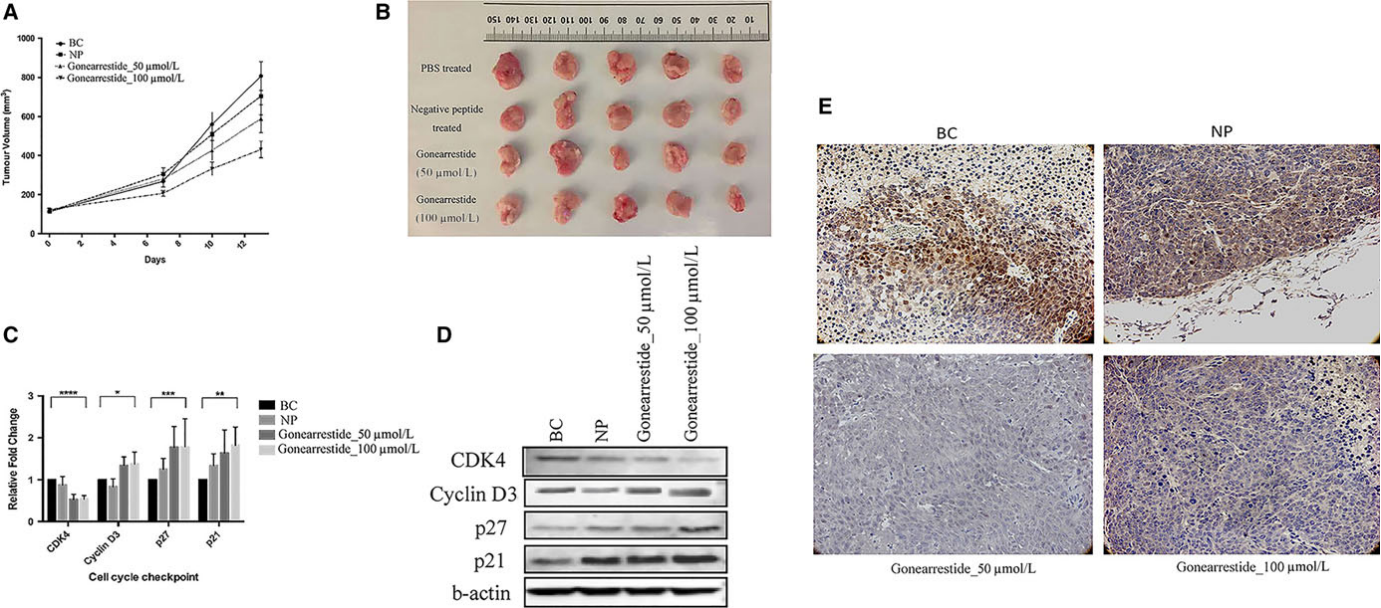
*In vivo* anticancer activity of Gonearrestide on HCT116 xenograft model (n = 5). A, Tumour volume growth curve after peptide treatment. B, Mice tumour tissues after sacrifice. C, Relative fold change of cell cycle‐related genes regulated by Gonearrestide on mice tumour. The levels of significance are: *P <* *.05; **P <* *.01; ***P <* *.001; ****P <* *.001. D, Western blot results of cell cycle checkpoints proteins regulated by Gonearrestide on mice tumour. E, Immunohistochemical analysis of paraffin‐embedded mice tumour tissue sections using cell cycle checkpoints antibody CDK4 (1:100), noted that CDK4 was dysregulated

The xenograft mouse model was employed to verify the robustness of the in vitro results. The data of resultant tumour volume with or without peptide treatments were shown in Figure 4A. The volume of implanted tumours was significantly reduced in a dose‐dependent manner of Gonearrestide. The negative peptide control group did not show any significance differences. These results confirmed that Gonearrestide could exert its anticancer activity in vivo. All mice were sacrificed at the end of the experiment and tumours were removed for further analysis (Figure 4B). The qPCR and Western blot data from the tumour tissues also showed that Gonearrestide could regulate cell cycle checkpoint proteins in vivo*,* and the trend was consistent with the in vivo results (Figure 4C and D). Immunohistochemical staining was also performed and showed that Gonearrestide could down‐regulate CDK4 in vivo as shown in Figure 4E.

### 3.7 Prediction of signalling pathways involved

**Figure 5:**
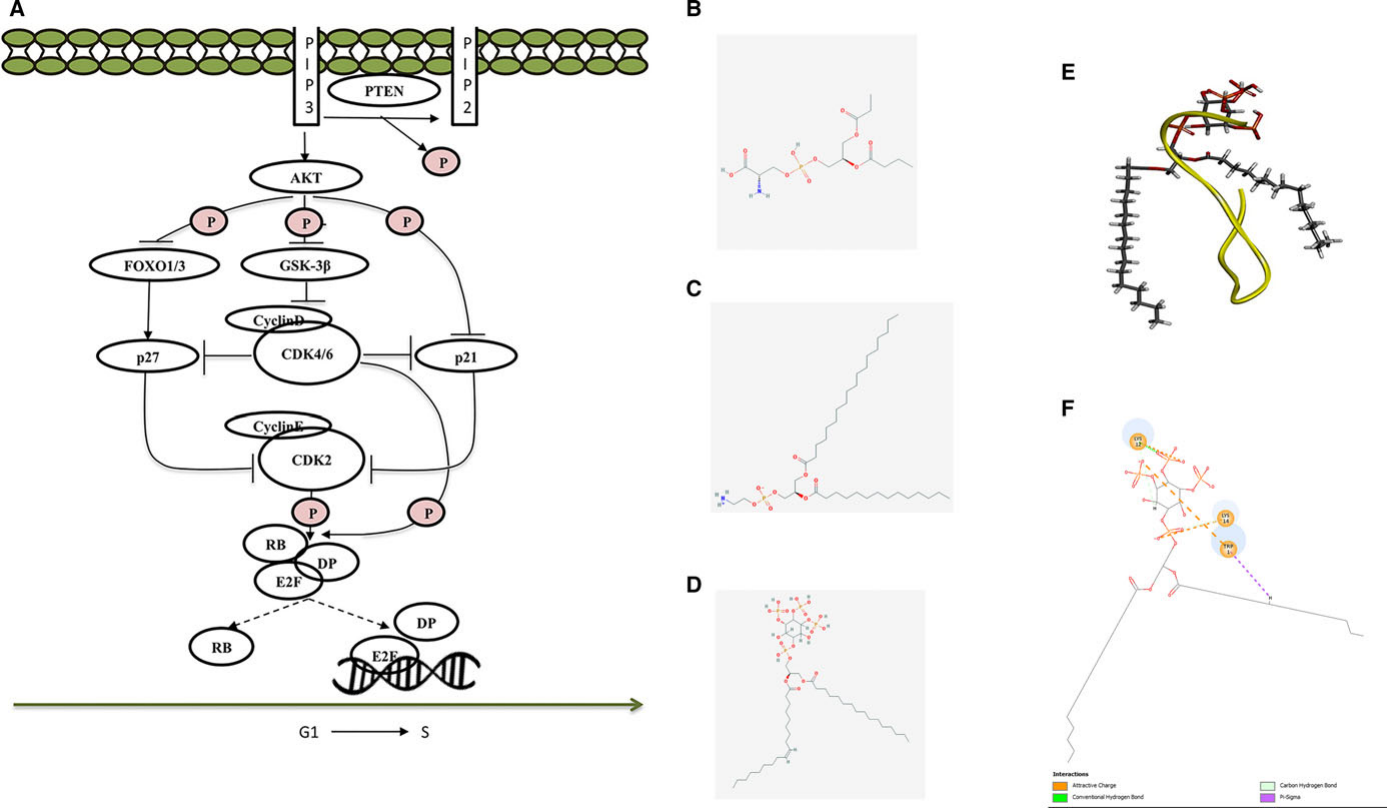
Predicted signalling pathways and potential binding site. A, Predicted signalling pathways involved in cell cycle progress of colon cancer HCT116 treated with Gonearrestide. Noted that the regulated genes were identified by RNA‐seq, among which p27, p21, CDK4/6 and Cyclin D3 were evaluated by Western bolt. B‐D, 2D structures of PS, PE and PIP3. E, Peptide‐lipid interactions between Gonearrestide and PIP3 lipid molecular generated through C‐DOCKER. F, 2D diagram of the peptide‐lipid interaction, which shows the electrostatic interaction between Gonearrestide and PIP3

Through combination of RNA sequencing data and biological in vitro*/*in vivo results, we hypothesized the signalling pathways involved in cell cycle progression, which were shown in Figure 5A. We believed that after Gonearrestide bound with the cancer cell membrane, cationic Gonearrestide could affect PIP3 by electrostatic attraction to its anionic trisphosphate group, resulting in the inhibition of the Akt pathway. PTEN, a lipid phosphatase that catalyses the dephosphorylation of PIP3 to produce PIP2, is a major negative regulator of Akt signalling. The inhibition of Akt was followed by the up‐regulation of FOXO1/3, GSK‐3β and p21, inducing the dysregulation of CDK4/6 and CDK2. The decline in CDK4/6 and CDK2 would inhibit the phosphorylation of retinoblastoma (RB) protein, which could release the transcription factor, E2F/DP, from the RB‐E2F/DP complexes, thereby promoting cells entry from G1 to S phase. Among all these genes, p27, p21, cyclin D3 and CDK4/6 were evaluated through Western blot assays, which further proved this prediction. On the other hand, after bioinformatics analysis, a series of biomarkers involved in peptide treatment related to colon cancer were also identified (Figure S5).

### 3.8 Hypothesis of potential binding site

It has been reported that phosphatidylserine (PS) and phosphatidylethanolamine (PE) can enhance membrane poration by a peptide with anticancer properties.^48^ As a lipid, PIP3 displays a similar 2D structure to PS and PE (Figure 5 B, C and D). Hence we hypothesized that Gonearrestide could bind to PIP3, resulting in the following reactions. Docking between the peptide and the lipid was performed to prove the concept through using C‐DOCKER in software Discovery Studio 2017 (Figure 5E). The 3D structure of PIP3 was constructed by homology modelling, and the 3D structure of PIP3 was transformed from the 2D structure downloaded from Pubchem. The 2D diagram of peptide‐lipid interaction was shown in Figure 5F. From the diagram, it demonstrated that the residues W1, K12 and K14 presented a charge attracting effect with PIP3, which further proved our hypothesis in Gonearrestide affects on PIP3 via electrostatic attraction to its anionic trisphosphate group.

## 4 DISCUSSION

Venom from animals has been proven to be a useful source of drug candidates in natural drug discovery to fight against diseases.^49^, ^50^ Especially for venom‐based peptide/toxin, it has been paved new insights into therapeutic and diagnostic potential for cancer treatment in the last decade.^51^, ^52^, ^53^, ^54^, ^55^, ^56^ In a leading edge review on cancer research by Hanahan and Weinberg, authors discussed six important hallmarks of cancer; and proposed an ideal anticancer drug should able to inhibit and/or block one or several cancer hallmarks.^57^ Recent studies have unveiled the interaction of venom peptides with some membrane receptor molecules, non‐receptor components, and/or extracellular matrix which could induce cancer hallmark dysregulation or inhibition.^57^ For instance, NN‐32, a protein toxin purified from *Naja naja* venom exerted a cytotoxic activity on MCF‐7 and MDA‐MB‐231 cells, and thereby induced cancer cell death directly.^51^ Another examples, WEV and sPLA_2_, isolated from snake venom from *Walterinnesia aegyptia* and *Vipera ammodytes meriodionalis,* respectively, induced apoptosis on a broad spectrum of cancer cell lines.^52^, ^53^, ^54^, ^55^ Furthermore, the combination of WEV and silica nanoparticles efficiently enhanced the in vivo suppressive effect in mouse models.^52^, ^53^, ^54^

To increase the identification of potential venom‐sourced anticancer peptides, a high efficiency and low‐cost screening platform is urgently required. In this study, a high‐throughput screening platform consisting of transcriptome and proteome sequencing is described and has been of proven efficacy. Based on the use of this platform, complete peptide libraries of venoms from the scorpions, *Androctonus mauritanicus* (AMa) and *Androctonus australis* (Egypt) (AAu), have been constructed. To confirm the validity of this platform, a traditional cloning approach was also applied in parallel. The traditionally‐derived cloning data provided evidence of the robust nature of the described high‐throughput screening platform. The coupling of transcriptomic and proteomic/peptidomic approaches using bioinformatics produced venom‐derived peptide panels, which could be used to explore the potential therapeutically useful peptides present in respective venoms.^58^

Within the scorpion peptide panels, bioinformatics filtration and MTT screening were initially applied to detect novel candidate anticancer peptides. As a result, peptide 13(Gonearrestide) was identified and found to have a potent anticancer activity against human colon cancer cell line HCT116 but with negligible observed cytotoxicity against normal human epithelial cells and erythrocytes. To study the anticancer mechanisms of Gonearrestide, RNA sequencing was used to detect the transcriptome responses in cells following Gonearrestide treatment. Transcriptome sequencing provided a number of advantages over conventional chemical screening strategies. This approach did not require prior identification of specific drug targets. This meant that, based on a multi‐target, pathway‐centric approach, any potential therapeutic targets could be assessed in the transcriptome landscape. Compared with phenotypic screening, transcriptome screening offers more specific gene expression responses providing crucial clues to potential molecular mechanisms.^32^ Thus, this approach was a powerful strategy to study the mechanisms of venom‐derived anticancer peptide drug actions.

The RNA sequencing results indicated that Gonearrestide could combine with cancer cell membranes followed by inhibition of cell proliferation through a cell cycle arrest effect. Although well‐designed in vitro, in vivo and ex vivo assays, the transcriptome‐centric prediction that Gonearrestide could arrest HCT116 cancer cells in G1 phase and inhibit tumour growth, was proven. The signalling pathway used involved effects on PIP3 and PTEN, resulting in the inhibition of the Akt pathway. PIP3 is a phospholipid that is bound within the layers of the cell membrane and after combining with the cell membrane, Gonearrestide can affect PIP3 by electrostatic attraction to its anionic trisphosphate group. PTEN catalyses the dephosphorylation of PIP3 to produce PIP2, which is a major negative regulator of Akt signalling. It is a tumour suppressor and the phosphatase activity of this protein may also be related to cell cycle regulation preventing cells growing and dividing too rapidly. From the previous anti‐proliferative screening results, it could also be seen that Gonearrestide showed a better anticancer effect on the PTEN wild‐type cell line, HCT116, when compared with the PTEN‐mutated cell lines, PC‐3 and SW872, with cell viabilities of 55%, 84% and 80%, respectively. The inhibition of Akt induced downstream dysregulation of CDK4/6 and CDK2, thereby promoting cell entry from G1 to S phase. In addition, the peptide‐lipid interaction generated by docking also proved the hypothesis that Gonearrestide could bind to PIP3 through electrostatic attraction and resulted in alerting the downstream cell cycle regulation.

Current results showed that Gonearrestide could inhibit cell proliferation in vitro through a cell cycle arrest effect and could reduce tumour growth in vivo, while possessing little observable cytotoxicity on human epithelial cells and erythrocytes. Hence, this peptide was deemed to have a great potential to be proceed to clinical trials as an anticancer drug candidate. To enhance the efficiency of anticancer peptide discovery, we emphasize the broad utility of our high‐throughput platform for venom‐derived peptide library construction and selection and also the interfacing of this with a transcriptome‐centric mechanistic study approach. The validity of this approach has been proven by well‐designed experiments described and employed in this study. Moreover, within the scorpion peptide panel, several additional anticancer peptides are awaiting further investigation.

## CONFLICTS OF INTEREST

The authors confirm that there are no conflict of interest.
