# Supplementary material for: Triggering of cancer cell cycle arrest by a novel scorpion venom‐derived peptide—Gonearrestide
Source: J Cell Mol Med. 2018 Jul 11;22(9):4460–73. doi: 10.1111/jcmm.13745 (PMC6111814; doi:10.1111/jcmm.13745)
Supplement: Supplementary file 1 [file JCMM-22-4460-s001.docx]

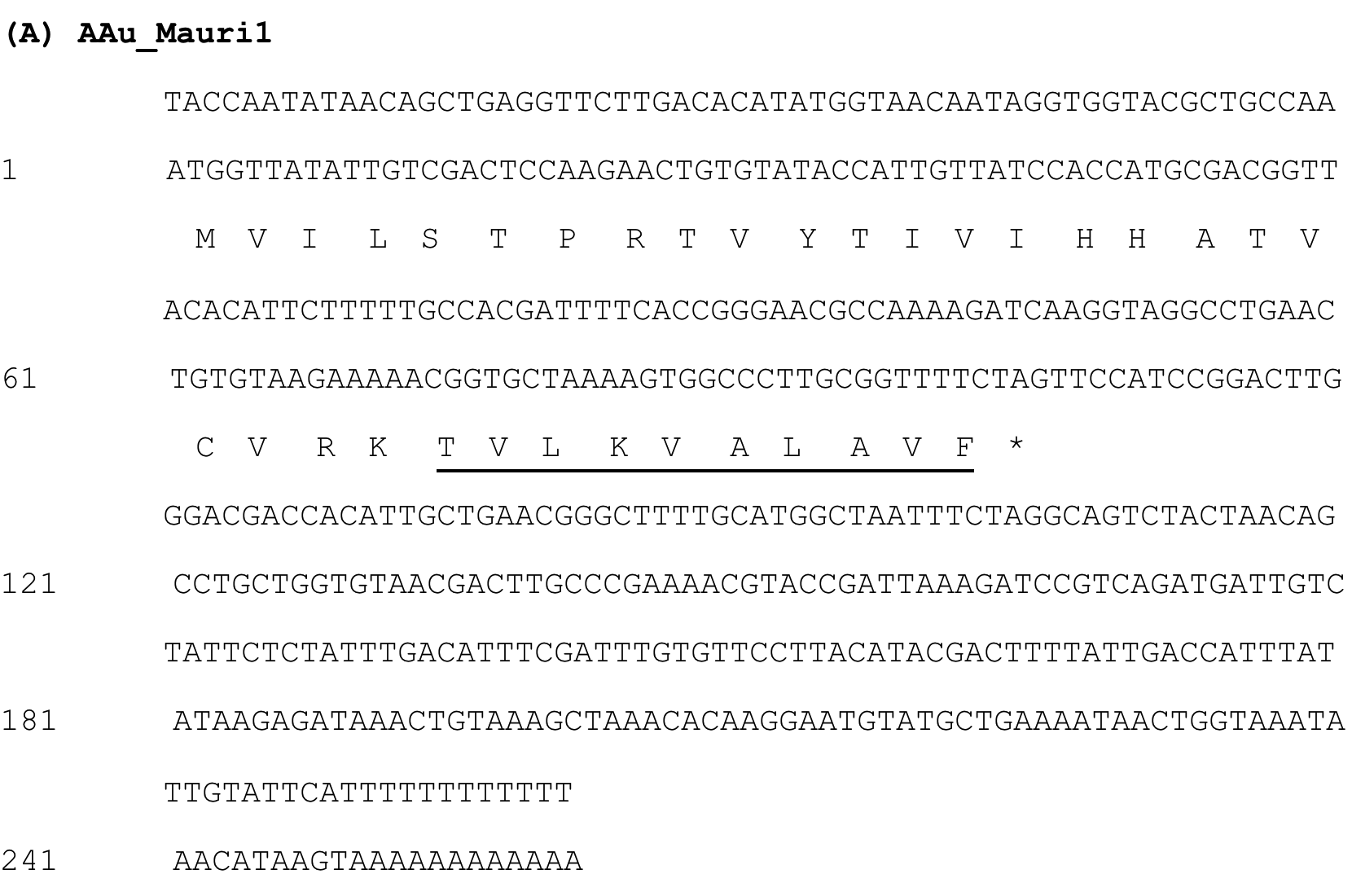


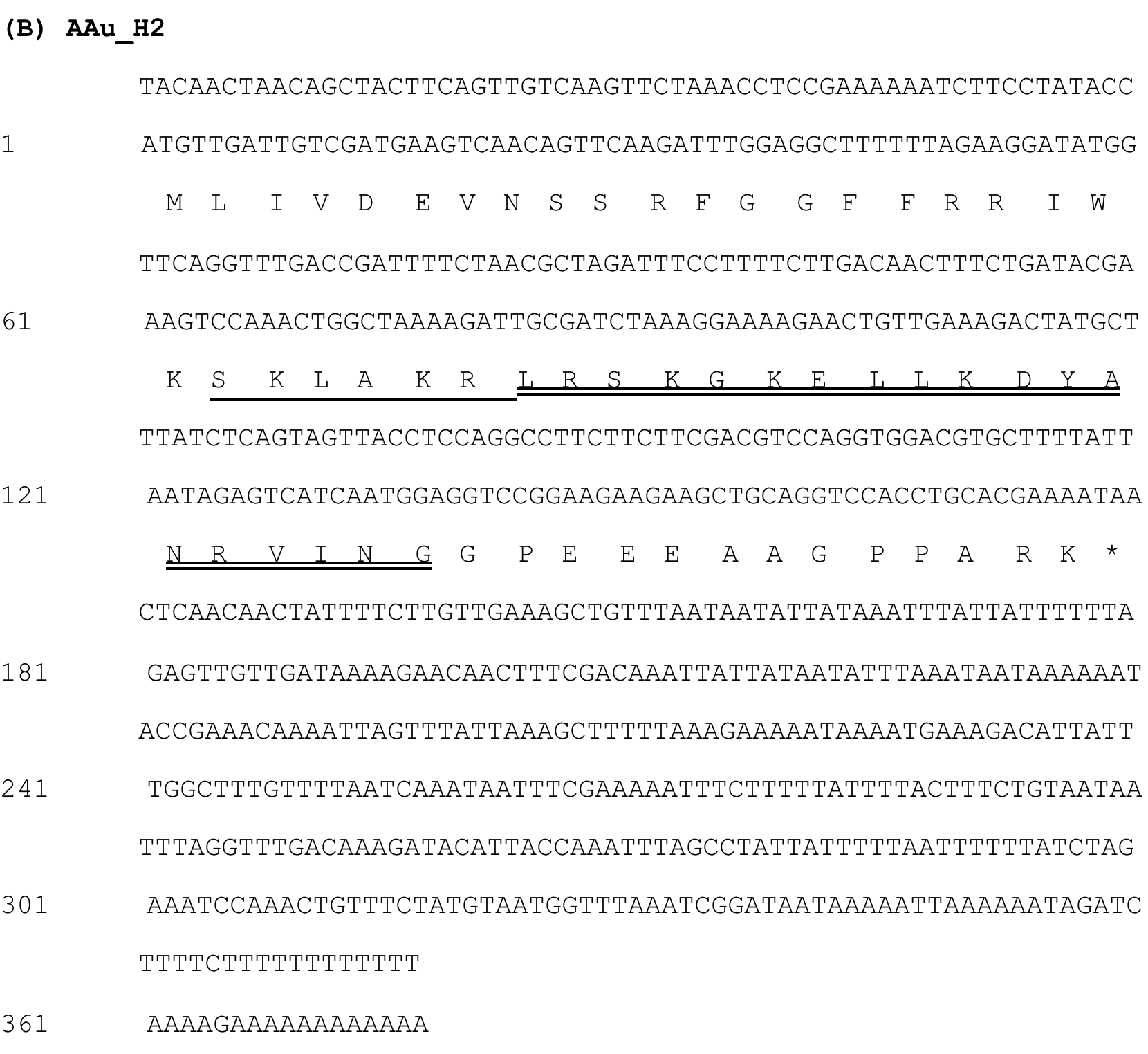


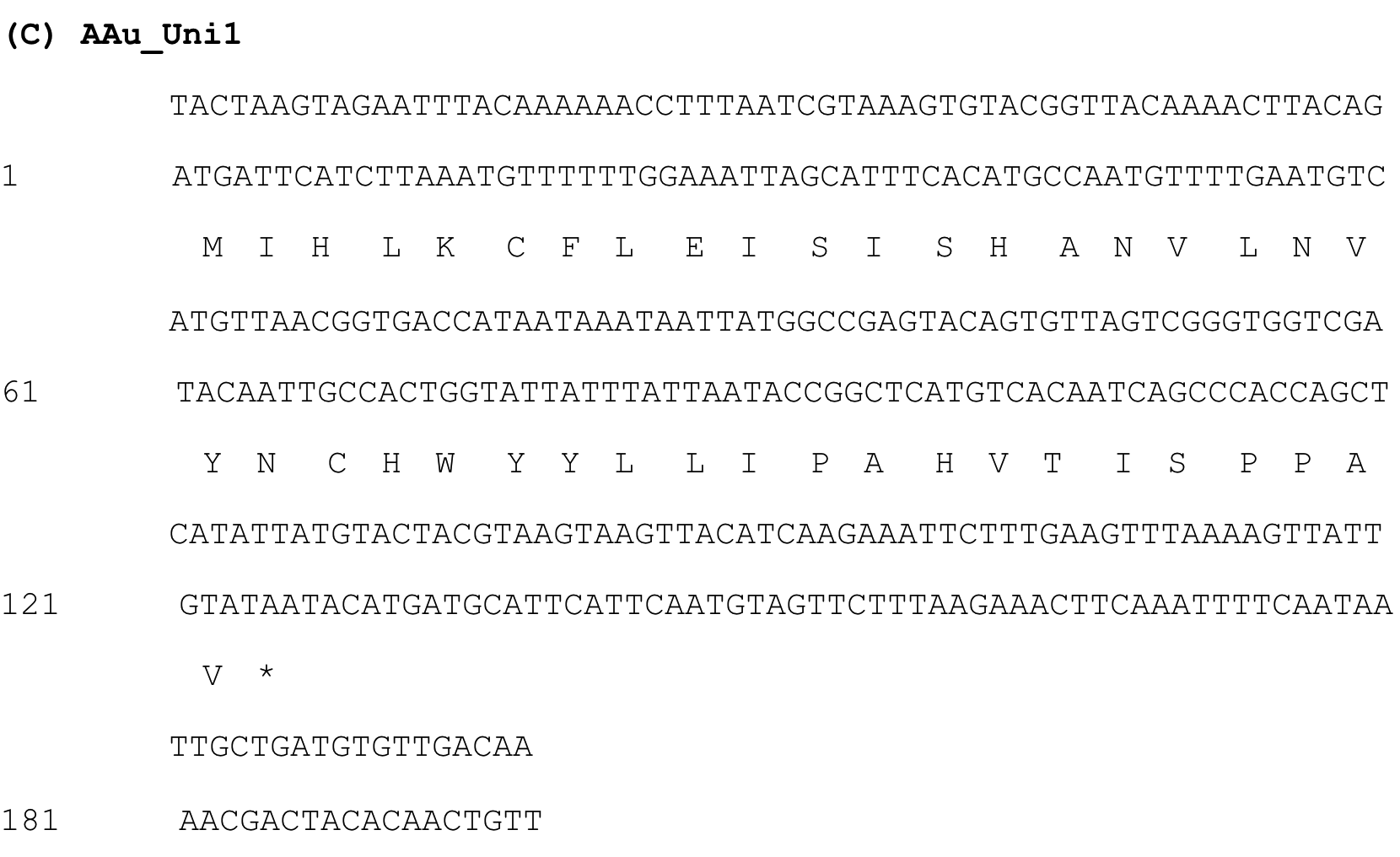


**Figure S1. Nucleotide and translated open-reading frame amino acid sequences of cloned cDNAs encoding the scorpion venom peptide precursors of (A) AAu_Mauri1, (B) AAu_H2, (C) AAu_Uni1.** Mature peptide sequences were single-underlined (1060,1140 and 2800) or double-underlined (2117), and stop codons were indicated by asterisks.

| (A) |
| --- |
| 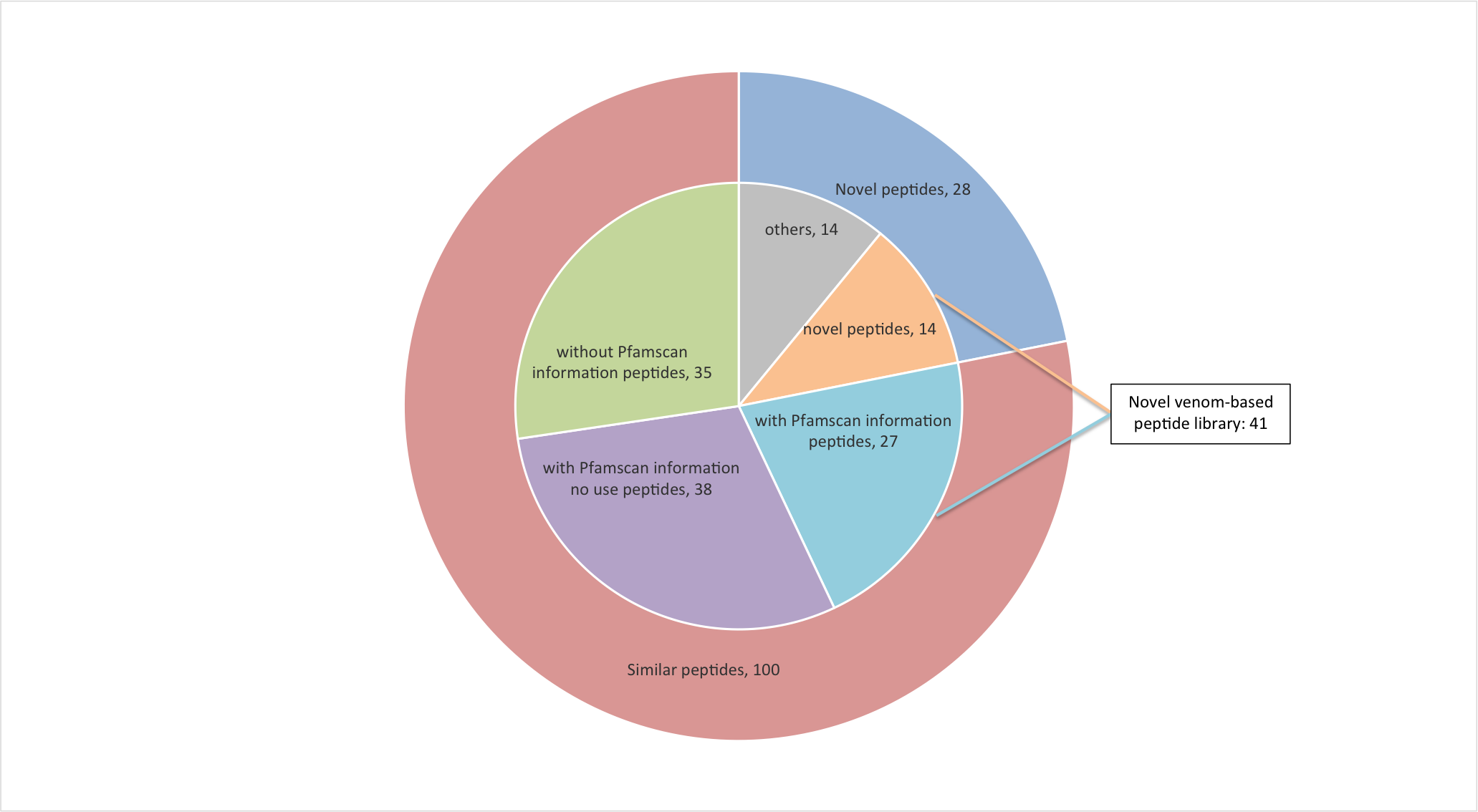 |
| (B) |
| 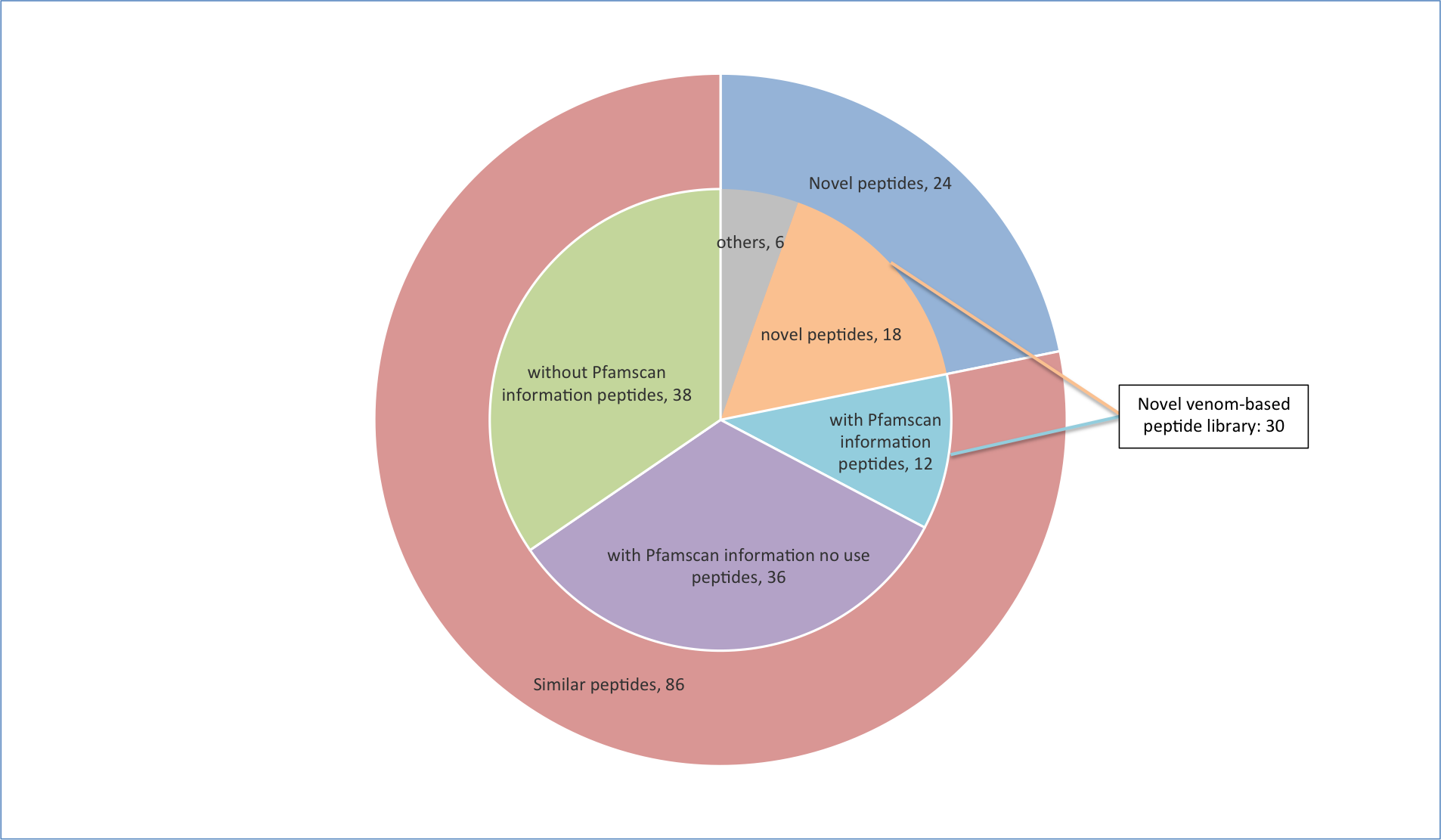 |
| **Figure S2. Bioinformatics filtration results of the two scorpion species.** (A) AMa. (B) AAu. |

| 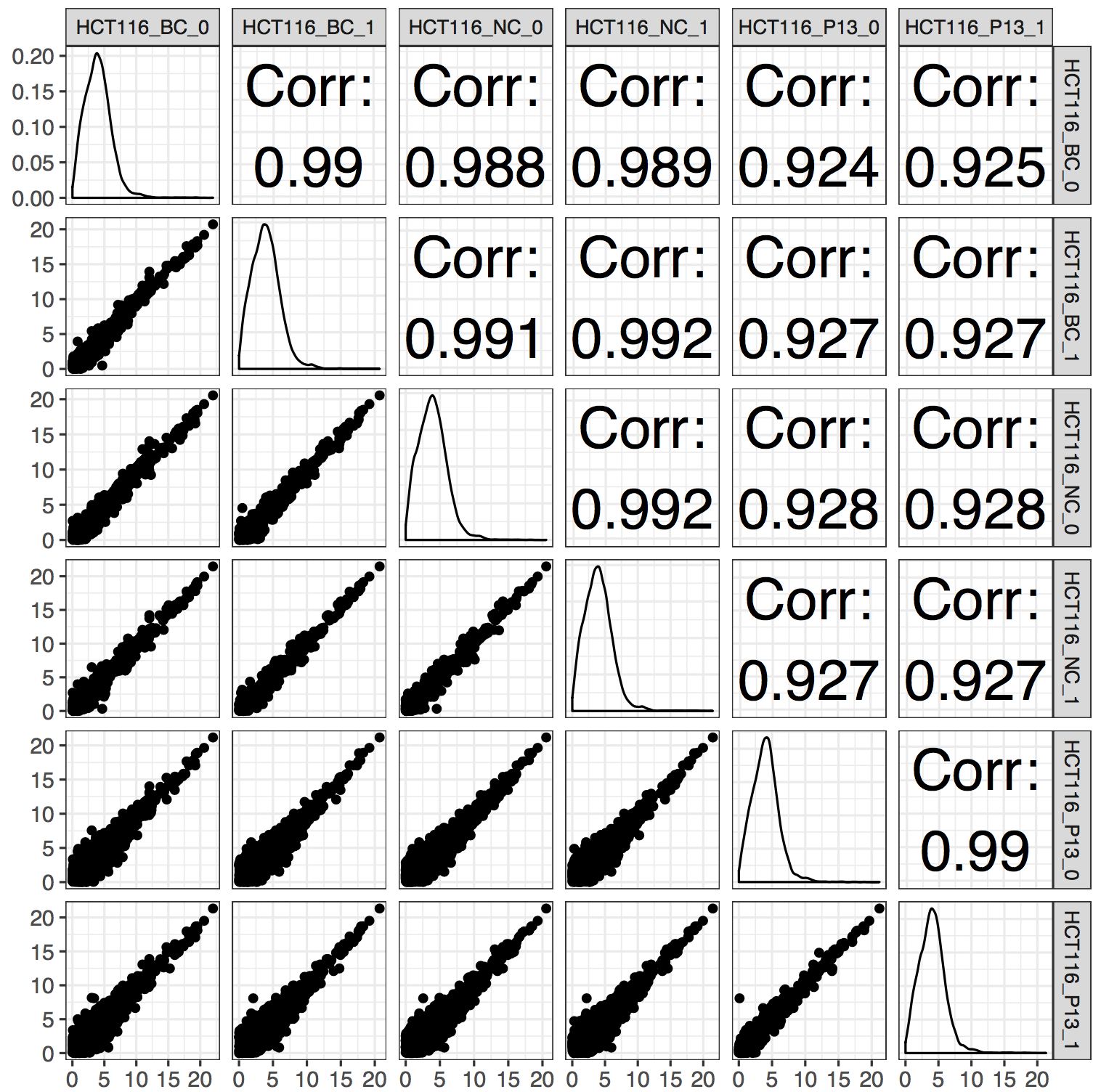 |
| --- |
| **Figure S3: Correlation plot of each sample.** |

| **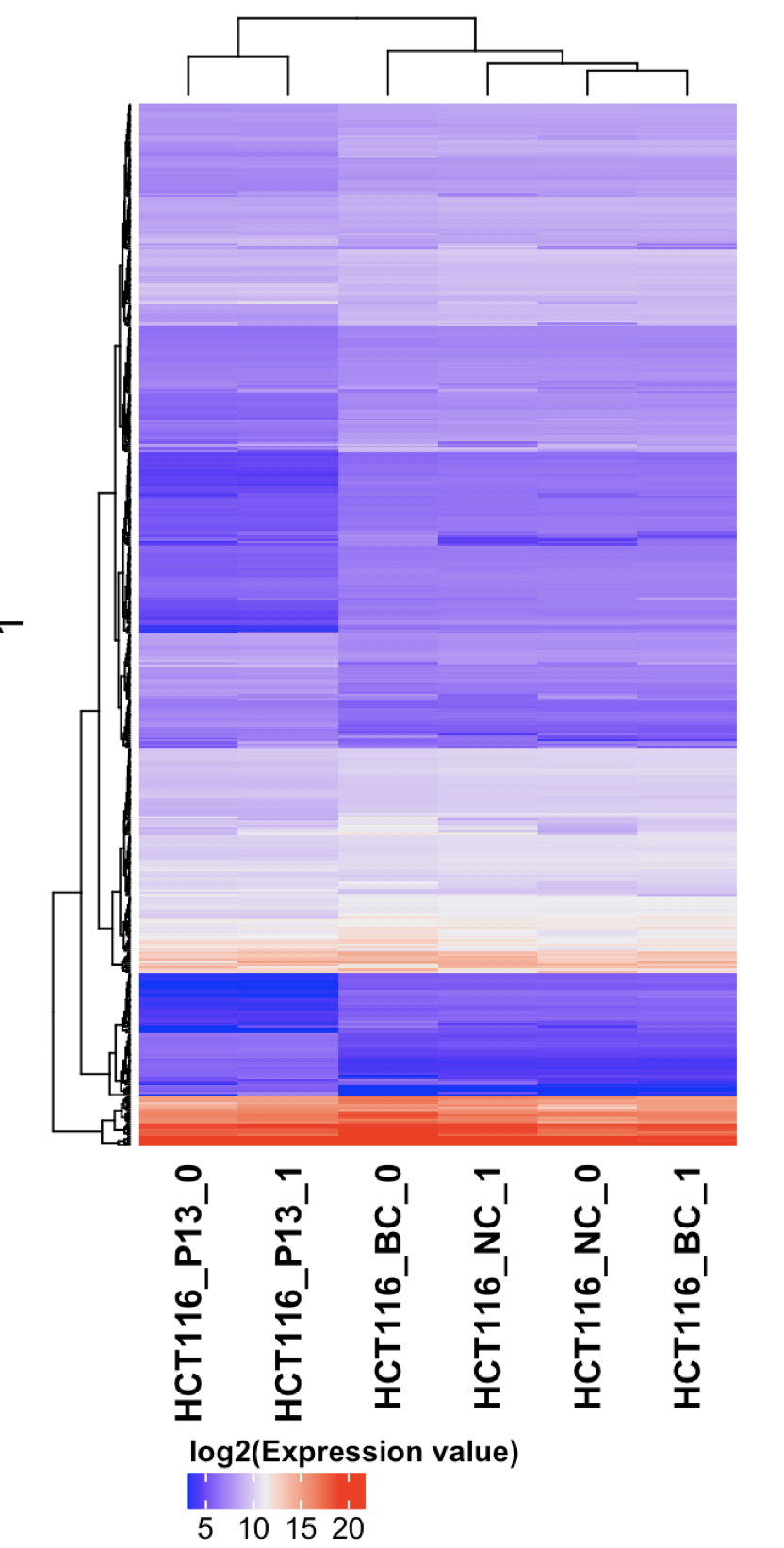** |
| --- |
| **Figure S4: Heat map of highly variable 500 genes.** |

| **** |
| --- |
| **Figure S5. Related biomarkers involved in peptide treatment in colon cancer. LFC is log2fold change: absolute (0.6).** |

**Table S1. Synthesized mature peptide sequences.**

| Peptides | RNA-seq name | Synthesized peptide sequences | Species |
| --- | --- | --- | --- |
| 1 | >c36414_g1_i1 | G K G K E F M A N I K E K L S G V K E | AAu |
| 2 | >c38296_g2_i1 | G R D G Y I V D S K N C V Y H | AAu |
| 3 | >c38296_g2_i1 | C W C V A L P D N V P I K D R S Y K C H S | AAu |
| 4 | >c38400_g1_i1 | A D A P G N Y P L D A R G K S Y Y C | AAu |
| 5 | >c38125_g1_i1 | C K C E G L P D D A K L W D Q T K K C | AAu |
| 6 | >c38381_g8_i1 | K L S A I I S K I R D E | AAu |
| 7 | >c38403_g1_i2 | N D A D K D E M Q S V Y Y G | AAu |
| 8 | >c38403_g1_i2 | N D A D K D E M Q S V Y Y G K A N D D N S | AAu |
| 9 | >c38403_g1_i2 | N D A D K D E M Q S V Y Y G K A N D D N S R G G K T S H R F | AAu |
| 10 | >c13273_g1_i2 | N D A D K D E M Q S V Y R G K A N D D N S R G S K T N H R F | AMa |
| 11 | >c31353_g1_i1 | A D N D D V G H K T I P F - NH2 | AMa |
| 12 | >c13404_g2_i1 | Y E L S A V L S K I R D E | AMa |
| 13 | >c40465_g1_i1 | W C Y K L P D R V S I K E K G R C N - NH2 | AMa |
| 14 | >c13395_g1_i1 | G L R E K H V Q K L V K Y A V P A G T L R | AMa |
| 15 | >c1463_g1_i1 | G K G K E F M A N I K E K L | AMa |
| 16 | >c13409_g1_i2 | A R D G Y V V H D G T N C K Y G C | AMa |
| 17 | >c13407_g2_i1 | C K D L P D N V P I K V Q G K C H | AMa |
| 18 | >c13255_g1_i1 | (Pyro-glu) Q I E T N K K C Q G G S C A S V C R | AMa |
| 1060 | AAu_Mauri1 | T V L K V A L A V F | AAu |
| 1140 | AAu_Mauri1 | p T V L K V A L A V F | AAu |
| 2117 | AAu_H2 | L R S K G K E L L K D Y A N R V I N G | AAu |
| 2800 | AAu_H2 | S K L A K R L R S K G K E L L K D Y A N R V I N G | AAu |
